# Ancient DNA Reveals Late Pleistocene Existence of Ostriches in Indian Sub-Continent

**DOI:** 10.1371/journal.pone.0164823

**Published:** 2017-03-08

**Authors:** Sonal Jain, Niraj Rai, Giriraj Kumar, Parul Aggarwal Pruthi, Kumarasamy Thangaraj, Sunil Bajpai, Vikas Pruthi

**Affiliations:** 1 Department of Biotechnology, Indian Institute of Technology, Roorkee, Uttarakhand, India; 2 CSIR-Centre for Cellular and Molecular Biology, Hyderabad, India; 3 Dayalbagh Educational Institute, Dayalbagh, Agra, UP, India; 4 Department of Earth Sciences, Indian Institute of Technology, Roorkee, Uttarakhand, India; Universitat Pompeu Fabra, SPAIN

## Abstract

Ancient DNA (aDNA) analysis of extinct ratite species is of considerable interest as it provides important insights into their origin, evolution, paleogeographical distribution and vicariant speciation in congruence with continental drift theory. In this study, DNA hotspots were detected in fossilized eggshell fragments of ratites (dated ≥25000 years B.P. by radiocarbon dating) using confocal laser scanning microscopy (CLSM). DNA was isolated from five eggshell fragments and a 43 base pair (bp) sequence of a 16S rRNA mitochondrial-conserved region was successfully amplified and sequenced from one of the samples. Phylogenetic analysis of the DNA sequence revealed a 92% identity of the fossil eggshells to *Struthio camelus* and their position basal to other palaeognaths, consistent with the vicariant speciation model. Our study provides the first molecular evidence for the presence of ostriches in India, complementing the continental drift theory of biogeographical movement of ostriches in India, and opening up a new window into the evolutionary history of ratites.

## Introduction

**Fig 1:**
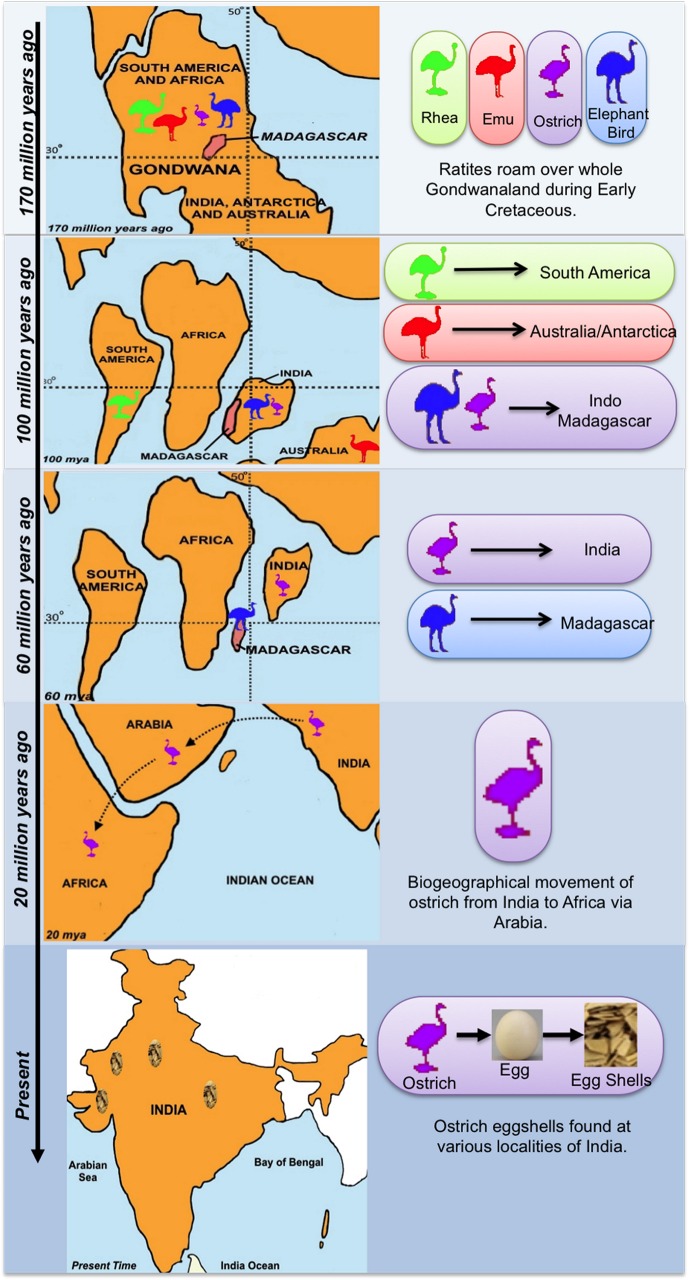
Continental drifting and vicariant speciation of ratites. Breakup of supercontinent Gondwanaland during Early Cretaceous and biogeographical movement of ratites to different continents.

The origin and evolution of ratites, the flightless birds, is widely attributed to the continental drifting of Gondwanaland [1]. One hundred and fifty million years ago, Gondwanaland was a consortium of South America, Arabia, Africa, Australia, Antarctica, India and Madagascar [2]. Initial breakup of this supercontinent during the Early Cretaceous, ~130 to 100 million years ago (Ma), separated Africa and Indo-Madagascar (Fig 1). Later, the remaining Gondwana landmass further split into South America and Australia/Antarctica [1]. During the Late Cretaceous (~80 Ma), Australia/Antarctica and Indo-Madagascar may have been connected through Kerguelen Plateau until India drifted further northwards following its break up from Madagascar [3]. This continental drift theory is complemented by the vicariant speciation that led to the evolution of ratites [1]. In the fractured Gondwana, rhea and emu migrated to South America and Australia, respectively, while elephant birds and ostrich existed together on Indo-Madagascar [2]. Continued northerly movement of India split the Indo-Madagascar landmass at ~88 Ma, with elephant birds remaining in Madagascar and ostriches in India. This biogeographical dispersion eventually led to hopping of ostriches in Africa through Eurasia via a land route around ~20 Ma [3]. The above studies impliy that elephant birds and ostriches should be the oldest ratite lineages. However, recent investigations by Mitchell et al, 2014 suggested that kiwis and elephant birds form the closest group, in contradiction with the continental drift theory [1].

Known occurrences of fossil eggshells of ratites in the Indian subcontinent range in age from the Middle Miocene to Late Pleistocene epochs [4–5]. Aigner reported ratite eggshells in 1935 from the Late Miocene Siwalik rocks of Dhokpathan near Hasnot in Punjab, Pakistan [6]. Evidence from the Siwalik formations revealed the presence of Struthionidae (ostrich family) from the Middle Miocene to Middle Pleistocene. No later records of the family are known from the Himalayan foreland but it is known to have existed in Central India [7]. Skeletal remains of the Siwalik ratites were identified as *Struthio asiaticus* (1871), whose eggshells differ in size from those of *Struthio camelus* [8–9]. Recently, AMS dating of eggshells confirmed the earliest evidence of ostriches on the Indian subcontinent and their expansion into Central and Western India, prior to 60 thousand years ago (ka) [5]. The eggshells can be identified on the basis of morphological characters such as eggshell thickness, pore complex shape, density and diameter [10–13]. Eggshells with struthinoid type pore patterns were found in younger Siwalik deposits ranging in age from 2.24 Ma to 0.5 Ma [14]. However, from Indian sites (Rajasthan, Uttar Pradesh, M.P., Maharashtra), fossil eggshells dating between >60000–18000 years ago have been identified as *Struthio* sp. [6,7,15]. This taxonomic identification is based on the pattern of decreasing pore diameter (1 mm), shell thickness (2.5 cm) and increasing pore density through time in comparison to other ostrich eggshells classified as *Struthio karingarbensis*, *Struthio daberasensis*, *Struthio kakesiensis* and *S*. *camelus* from Late Miocene and Pliocene of Namibia and East Africa [5]. Ostrich eggshells with a mean shell thickness of 2.54mm, identified as *S*. *asiaticus*, were found in Ahl al Oughlam, Morocco from Late Pliocene deposits. This shell thickness overlaps with the range observed in *S*. *daberasensis*, but the latter has a greater pore density comparable to *Struthio camelus*, thus following the general African trend [16]. The Late Pleistocene ostrich populations in India also show decreased shell thickness and high pore density similar to the African taxa. However, assignment of the Indian material to the African species based on morphological similarity alone may be problematic as this similarity may be a result of similar paleoenvironmental conditions [17–20]. DNA based species identification offers an alternative tool for authenticating the presence of ostriches in Indian peninsula.

**Fig 2:**
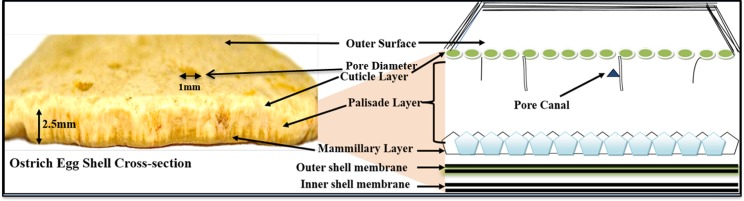
Ostrich Eggshell. Pictorial view and radial cross section showing various layers, shell thickness and pore diameter.

Recently, avian fossil eggshells have been characterized as a potent source of preserved aDNA biomolecules [21]. The ultrastructure of eggshells shows a characteristic morphology consisting of several layers (mammillary layer, palisade layer and cuticle layer) beginning from the inner to outer surface (Fig 2). The matrix of the avian eggshell is porous, an ordered and heterogeneous complex, which is coupled with an extracellular structure composed of mineralized and non-mineralized regions having calcium carbonate (CaCO_3_) (calcite 97%) and 3.5% organic matrix [18,22–23]. The intracrystalline organic matrix of avian eggshells prevents diffusion losses and isotopic exchange of organic constituents [19,21,24]. This structural hallmark provides an external skeletal support to the developing avian embryo and controls gases, water exchange as well as guards against microbial interventions and physical stresses [21, 25–27]. This structure also imparts stability as well as excellent mechanical strength to the eggshell, besides providing antibacterial and antifouling properties thus enhancing the preservation potential of biomolecules in eggshells [25,28].

In this study, we examined the extent of aDNA preservation in eleven fossilized avian eggshell samples collected from eight archaeological sites in central and western parts of India (S1 Fig). The eggshells used in this investigation have survived harsh environmental conditions [5] and have been dated to Upper Palaeolithic (25,000 to 40,000 years B.P) based on the stone tool technology [6,29–30] and radiocarbon dating at Groningen University Lab, Germany [31,32]. Some of these eggshells are engraved into finished or unfinished beads [33–35]. The perforated beads belong to archaeological assemblages that correspond to the same time period when modern human populations would have dispersed out of Africa [36]. The presence of DNA hotspots in the mammillary layer was detected with DNA binding fluorescent dye Hoechst 33342 and imaged through Confocal Laser Scanning Microscopy. DNA was isolated, quantitized using Bioanalyzer 2100 (Agilent) and a 43 bp sequence of the mitochondrial coding region (16S rRNA) was amplified and sequenced from one of the samples using species-specific primers. The DNA sequence have been submitted to NCBI GenBank (accession number KU251475).

## Materials and Methods

### Materials

Fossilized eggshell samples used in this study came from the excavation sites and personal museum collections of our team members. These eggshell fragments were recovered at excavation sites at Bundi (Rajasthan state, western India) and from the Pleistocene surface deposits of Gujarat, western India. Eggshell fragments, collected from eight different sites in India were analyzed (Table 1). These samples have been assigned to the genus *Struthio* on the basis of morphological characteristics, especially thickness and pore pattern on the eggshells [6, 29]

**Table 1:** Sample collection sites and their respective states.

S.No.	Site	Area	State & GPS Co-ordinates	Catalogue No.	Dates	Method	Reference
**1**	Chavni Baroda	Bundi	**Rajasthan** (25° 26′ 24″ N, 75° 38′ 24″ E)	SJ/CB/001	-	-	-
				SJ/CB/002			
**2**	Anjar	Kachchh	**Gujarat** (23° 6′ 48.32″ N, 70° 1′ 39.88″ E)	SB/AN/003	>25000	**C**^**14**^	**Sahni et al. (1989)**
**3**	Chandresal- 1	Kota	**Rajasthan** (25° 10′ 48″ N, 75° 49′ 48″ E)	GK/CH1/004	38900±**750**	**C**^**14**^	**Mishra (1995)**
				GK/CH1/005			
**4**	Chandresal -2	Kota	**Rajasthan** (25°13'34.1"N 75°55'34.7"E)	GK/CH2/006	36500±**600**	**C**^**14**^	**Agarwal et al. (1991)**
				GK/CH2/007			
**5**	Nagda	Chambal	**Rajasthan** (25°10'53.3"N 75°48'45.5"E)	GK/NA/008	>31000	**C**^**14**^	**Mishra (1995)**
**6**	Runija	Ujjain	**Madhya Pradesh** (23°09'39.4"N 75°16'02.6"E)	GK/RU/009	>25000	**C**^**14**^	**Mishra (1995)**
**7**	Khajurna	Bijora	**Madhya Pradesh** (26°44'33.6"N 78°47'45.6"E)	GK/KH/010	>25000	**C**^**14**^	**Mishra (1995)**
**8**	Ravishankar Nagar	Bhopal	**Madhya Pradesh** (23°15'34.8"N 77°24'24.9"E)	GK/RN/011	>25000	**C**^**14**^	**Mishra (1995)**

### Confocal Laser Scanning Microscopy

To determine the presence and location of DNA preserved in ratites eggshells, we used a confocal scanning microscope. The samples were incubated in square chambered plates with DNA binding fluorescent dye, Hoechst 33342 (1mg/ml) (Sigma, USA) at room temperature for 15 minutes and imaged under Leica TCS SP5 AOPS Confocal system. Images were captured (405 nm) using 10X and 40X (1.25 oil immersion) objectives, while 3-D sections snapshots were taken using Galvo-Z stage for Z sectioning. Cross sectional images of eggshells obtained were analysed using LASAF LITE software. Unstained eggshell samples were used as control.

### Quality control

A major concern throughout the present molecular analysis was contamination by any exogenous DNA. The DNA extraction was performed in a dedicated ancient DNA facility at CCMB, Hyderabad, India. This lab has already carried out investigations on human aDNA [37]. Disposable gloves, full body suits and face masks were used throughout the sample processing. The ancient DNA lab functions under positive pressure created through 5μM HEPA filters and is UV irradiated every night.

All samples and reagents were exposed to UV light. All equipments and benchtops were regularly cleaned with 20% bleach solution. Disposable laboratory wares, gloves and laboratory coats were used during the experiments. For each sample two independent extractions were prepared and done by two independent researchers. Every day, a single sample was extracted using controls. Controls consisted of PCR reaction mixtures without the sample. All the parameters were followed in order to avoid the risk of contamination [38–40].

### Sample preparation and DNA isolation

Eggshell samples were wiped with 5% hypochlorite solution followed by surface cleaning using 70% alcohol. A small piece of eggshell was cut from the source eggshell using Dremel MultiPro tools and around 700-800mg of powder was collected per sample. DNA extraction was performed on the powdered eggshell samples using a published protocol with slight modification [37,41]. Briefly, one gram of powder was incubated in a digestion buffer (700 mL per sample) containing final volumes of 0.47 M EDTA (pH 8.0), 20 mM Tris (pH 8.0), 1% Triton X-100, 10 mM Dithiothreitol (DTT) and 1 mg/mL proteinase K for up to 24 hours, followed by a final heating at 92°C. The heating aids in solubilisation of calcite and in releasing the DNA from crystalline matrix. The heating step was modified to prevent the complete denaturation of DNA. The solution was then concentrated with 30,000 KDa MWCO columns (Millipore) and purified using commercial silica spin columns (Qiagen) with slight change in manufacturer’s protocol i.e incubating the elution step for 5 minutes at 55 degree centigrade.

### Ancient DNA quantity

Eluted DNA samples were quantified using the high sensitivity chip on the Bioanalyzer 2100 (Agilent) and prepared for PCR amplification and sequencing. The quantity of total DNA was estimated to be close to the lowest detection limit of the Bioanalyzer.

### PCR amplification and sequencing

For amplification of 16S rRNA region and control region of mitochondrial DNA, primers were designed as shown in Table 2. Same fragments from each sample was amplified in three DNA extracts to validate results. Annealing temperature was changed and the annealing time was extended. Primers were designed using Primer 3 plus and using mtDNA reference sequence of *Struthio camelus*. As DNA quantity was extremely low, Titanium Taq DNA Polymerase (Roche) was used, which is mostly used for High-Throughput genotyping using MassArray System (Agena Biosciences).

**Table 2:** Primer sequences used for PCR and sequencing of 16S and Hypervariable regions of mitochondrial DNA.

Primer name	Sequences	Gene
2F	5'GTACCGCAAGGGAAAGATGA3'	16S rRNA
2R	5'GGATGGCAAGCTTAAATTCG3'	
4F	5'GGGGGTTACCCTCTAATGGA3'	16S rRNA
4R	5'AGCTGGTTGCCTGTGAAAAG3'	
7F	5'CGACTCAGGAGCGCCTATTA3'	16S rRNA
7R	5'TGGCTGAAGGCTATGTTTTTG3'	
1CF	5'TCGCGATTAAGAGGGACAAT3'	Hypervariable Region
1CR	5'TCCATTCACGTTCCCCTTTA3'	

PCR reaction was set up in 5 μl total volume per reaction. The reaction mixture contained 2 μl of eluted DNA, reaction buffer supplied by the manufacturer (Agena Biosciences), dNTPs at 200 μM each, Taq polymerase (Roche) at 0.1 unit (0.02 μl at 5 U/μl, primers at 200 nM and MgCl_2_ at the final concentration as mentioned in the protocol. PCR cycling conditions for Titanium Taq DNA polymerase (Invitrogen) included: initial heating to 95°C, followed by 45 cycles of 94°C for 20 sec, 56°C for 30 sec, and 72°C for 1 min, and a final step of 72°C for 3 min. After PCR, the products were checked on 3% agarose gel (Sigma Aldrich) using 50bp DNA ladders (NEB) and the expected product sizes were confirmed. PCR products were purified using Shrimp Alkaline Phosphatase (SAP) from Agena Biosciences as per the manufacturer protocol.

### Cloning

PCR products were cloned using the pMOS Blunt-ended PCR cloning kit (GE Healthcare, USA). PCR products were ligated to pMossBlue dephosphorylated blunt vector as per the manufacturer protocol. 2 μl of ligated product and DH5α competent cells were used for transformation. Sample to vector ratio was adjusted to optimize insert-specific transformed cells. Transformed cells were spread on LB agar media and plate was incubated at 37°C overnight. For screening, white blue selection was made, Almost half of the colonies were directly used for colony PCR and for the remaining half plasmid DNA was isolated using plasmid isolation kit (Qiagen).

### DNA sequencing

The purified plasmids were sequenced using M13 forward primer as well as insert specific forward and reverse primers using Sanger’s dideoxy chain terminator cycle sequencing method. In case of colony PCR, PCR products were purified by treating with Exonuclease I and Shrimp Alkaline Phosphatase (ExoSAP-IT®; USB Corporation, Cleveland, Ohio, USA) and incubated at 37°C for 15 min. then at 80°C for 15 min. Purified PCR products (1.0 μl) were subjected to sequencing reaction, by adding 0.65–2.0 pmoles of the primer and 3.2 μl of BigDye^TM^ (Applied Biosystems, Foster City, CA, USA; containing fluorescently labeled ddNTPs and unlabeled dNTP). The sequencing PCR conditions include: 30 cycles of 94°C for 10 sec, 55°C for 5 sec and 60°C for 4 min. The PCR product was precipitated using 3 M sodium acetate (pH 5.2) and ethanol, and centrifuged at 4,000 rpm at 4°C for 15 min. The pellet was washed in 70% alcohol, air dried and suspended in 10 μl of HiDi^TM^ formamide (Applied Biosystems, Foster city, USA) and loaded on to ABI 3730 Automated DNA analyzer, after denaturation at 94°C for 10 min. The samples were run using POP-7^TM^ polymer and analyzed using ‘Sequencing Analysis’ software (Applied Biosystems, Foster city, USA) and MEGA 6.

### Negative Control Amplification

Deionized water was used as negative controls for the PCR amplification instead of the DNA extracts, following the same protocols and using the same reagents. No amplification of the negative control reactions confirmed the absence of lab-based contamination.

## Results

Eleven fossil samples from India, collected from eight archaeological sites (Table 1) and from the personal collections of palaeontologists, were investigated to identify the extent of DNA preservation in these fossil avian eggshells and for DNA based species identification. To maximize the DNA recovery from fossil eggshells, it was important to determine the physical location of DNA and to microscopically identify its presence in the inner, outer or calcified layers of the eggshell.

### CLSM of eggshells

**Fig 3:**
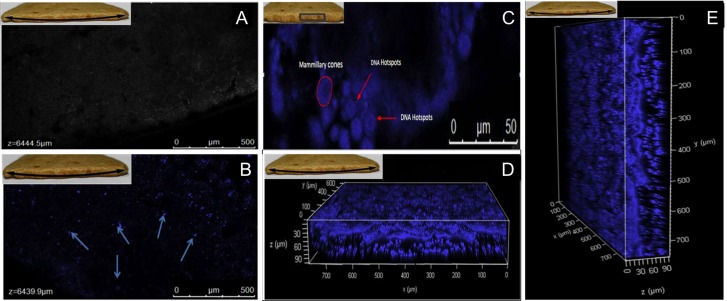
**Confocal images of ratite eggshell** (A) Control sample, without staining showing no fluoroscence (B) Sample stained with Hoechst 33342, displaying the DNA distributed throughout the matrix. Arrow indicates the presence of hotspots in fossilized eggshells (C) Fluorescently labelled DNA can be observed on the periphery of mammillary cones. Scale Bar 50 μm. (D) Three-dimensional imaging of inner layers of eggshell shows DNA distributed throughout the matrix as observed through fluorescent hotspots in horizontal view (E) Vertical view [Inset- Picture of eggshell. Marked area was observed under CLSM].

DNA in ratite eggshells is distributed uniformly throughout the eggshell matrix but may be concentrated around the periphery of the mammillary cones [21]. Confocal imaging of the avian eggshells studied here demonstrated the distribution of DNA throughout the eggshell matrix, as evident from the DNA hotspots observed after staining with Hoechst 33342, a fluorescent dye used to stain DNA (Fig 3A and 3B). The control image of unstained eggshells did not show any hotspots, confirming that the fluorescence observed in sample images was due to the binding of dye with DNA. The inner surface images of the eggshell fragments showed the concentration of DNA hotspots on the periphery of mammillary cones (Fig 3C). Three dimensional images captured using Z stage at different angles clearly validate the presence of DNA hotspots distributed uniformly throughout the matrix (Fig 3D and 3E).

### DNA extraction and species determination

**Fig 4:**
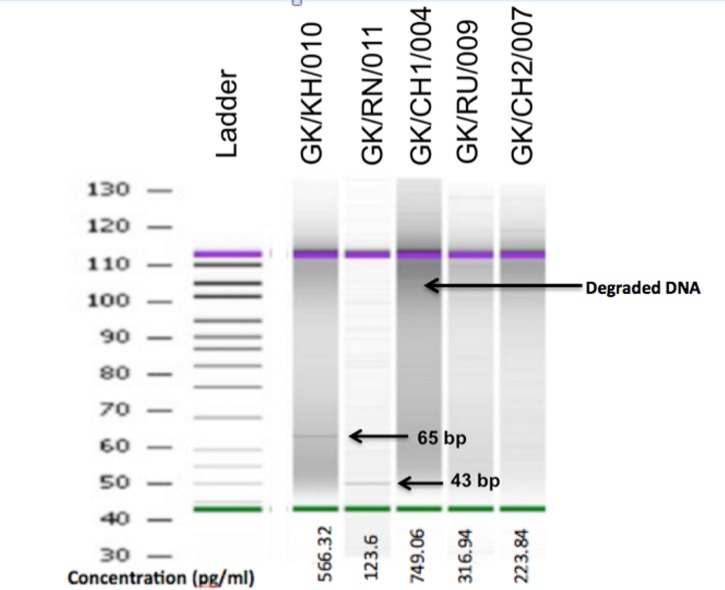
Quantification of fossilized DNA. Bioanalyzer image showing bands of 65 bp and 43 bp in GK/KH/010 and GK/RN/011 respectively. Concentration of DNA in pg/ml of five samples, calculated by bioanalyzer.

Five samples (GK/RN/011, GK/RU/009, GK/CH1/004, GK/KH/010 and GK/CH2/007), from five different sites, yielded aDNA (Table 3). Since the copy number of DNA is traditionally low in ancient samples and aDNA is fragmented in nature, we optimized the amplification protocol targeting the mitochondrial hypervariable region (HVR) and 16S rRNA gene by using PCR primers designed to amplify short fragments, using multiple aliquots of the DNA extracts as template for the PCR reactions. The extractions and PCR set up were done at CCMB, Hyderabad, India in facilities dedicated to the analysis of low copy number template. The DNA extracts were visualized on the Bioanalyzer 2100 (Agilent). Bioanalyzer revealed a concentration of <100pg/ml in the five samples GK/RN/011 (123.6 pg/ml), GK/KH/010 (566.32 pg/ml) GK/CH1/004 (749.06 pg/ml), GK/RU/009 (316.94 pg/ml) and GK/CH2/007 (223.84 pg/ml). Quantification of DNA extracted from the fossil eggshell samples showed a strong band around 45 bp in GK/RN/011 and 65bp in GK/KH/010 (Fig 4). GK/RN/011 was further used for phylogentic analysis. Bioanalyzer images of other samples showed multiple bands and smears which could be due to degraded nature of aDNA. The extracted and amplified DNA were further cloned using pMOS blunt ended PCR cloning kit and multiple clones were sequenced.

**Table 3:** List of eggshell specimens from India with associated genes and predicted taxon.

Site	Specimen ID	Dates	Predicted taxa	Gene	Amplicon site	Fragment length (bp)	E value (NCBI)	Identity	Taxon	Common name
Ravishankar Nagar	GK/RN/011	>25000	*Struthio Camelus*	16s RNA	1255–1298	43	2e-07	92%	*S*. *Camelus*	Ostrich
Runija	GK/RU/009	>25000	*S*. *Camelus*	16s RNA	1931–1954	23	6e-09	100%	*S*. *Camelus*	Ostrich
Chandresal-1	GK/CH1/004	38,900 ± 750 BP	*S*. *Camelus*	16s RNA	4859–4882	23	2e-07	96%	*Apteryx mantellis*	Kiwi
Khajurna	GK/KH/010	>25000	*S*. *Camelus*	HVR	15586–15606	20	0.019	89%	*S*. *Camelus*	Ostrich
Chandresal-2	GK/CH2/007	36,550 ± 600 BP	*S*. *Camelus*	HVR	15707–15724	17	1e-05	87%	*S*. *Camelus*	Ostrich

Dates according to Kumar et al, 1988; 1990

Taxon based on the closest GenBank BLAST match

Predicted taxa on the basis of eggshell morphology and location

HVR Hypervariable Region

**Fig 5:**
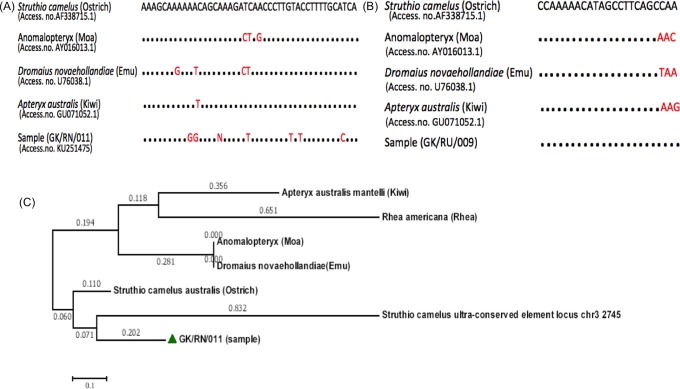
Alignment of partial 16s rRNA gene sequences of samples and ratites and evolutionary relationships of taxa. (A) and (B) Alignment of ratites sequences and sample GK/RN/011 and GK/RU/009. Dots signify identity to the *Struthio camelus* sequence. (C) The evolutionary history was inferred using the Neighbor-Joining method [42]. The bootstrap consensus tree inferred from 1000 replicates [43] is taken to represent the evolutionary history of the taxa analyzed [43]. The evolutionary distances were computed using the Maximum Composite Likelihood method [44] and are in the units of the number of base substitutions per site. The analysis involved 7 nucleotide sequences. The codon positions which were included: 1st+2nd+3rd+Noncoding. All positions containing gaps and missing data were eliminated. There were a total of 87 positions in the final dataset. Evolutionary analyses were conducted in MEGA6 [45].

A fragment length of 43 bp and 23 bp, corresponding to GK/RN/011 and GK/RU/009 respectively, was obtained from 25,000 years old samples collected from Ravishankar Nagar (GK/RN/011) and Runija (GK/RU/009), respectively, which showed 92% and 100% similarity with *Struthio camelus*. Alignments of partial 16S rRNA gene sequence of ratites (obtained from NCBI) and samples GK/RN/011 and GK/RU/009 were done (Fig 5). Other samples GK/CH1/004, GK/KH/010 and GK/CH2/007 collected from Chandresal 1, Khajurna and Chandresal 2 also attained a fragment length of 23 bp, 20 bp and 17 bp, respectively. Obtained sequences were compared with published partial mitochondrial sequences on NCBI. BLAST (NCBI) and multiple sequence alignment showed 92% similarity with *Struthio* species.

### Phylogenetic analysis

To assess the phylogenetic relationship of our DNA sequences, a comparative study with published avian mtDNA sequences was done and the phylogenetic tree was reconstructed (Fig 5) using MEGA 6.06. The phylogenetic analysis was based on maximum parsimony, neighbour joining and maximum likelihood [42–45]. These three methods are based on different assumptions and, thus, can be used complementarily to confirm the obtained phylogeny. The neighbour joining method was further extended and bootstrap consensus tree was inferred from 1000 replicates. The sample sequence shows phylogenetical similarity to the ratites family, forming a sister group with S. *camelus* (Fig 5). Phylogenetic tree showed the ostrich clade as basal to studied palaeognaths, which is consistent with the vicariant speciation model and other molecular phylogeny studies on ratites [1,3]. The estimates of evolutionary divergence between sequences were conducted in MEGA 6 (S1 Table) using the Maximum Composite Likelihood model.

## Discussion

The breakup of the supercontinent Gondwanaland during the Early Cretaceous resulted in the vicariant speciation of ratites, the flightless birds, to various countries that we know today as South America, Australia, New Zealand, India and Madagascar. The continental drift theory implies that ostriches migrated to India from other landmasses, consistent with the geographical distribution of fossil records of ratites. The fossil eggshells used in this study date to 25–40 ka and morphologically characterized as belonging to *Struthio camelus* [6,30]. Ostriches were believed to be extinct in India during the Late Pleistocene but our investigation reports the first genetic evidence of their presence on the subcontinent. Fossil eggshells have been reported as being more conducive to biomolecular preservation compared to fossil bones due to their intracrystalline structure, which minimizes microbial contamination [25,28]. This study is significant as it is the first report, to the best of our knowledge, of long-term DNA preservation in fossil eggshells collected from tropical environments with heterogeneous climatic conditions as those encountered in India. aDNA survival in fossils is usually determined by the thermal age theory[46]. This theory requires specific parameters including temperature, pressure and climatic conditions of fossil locations. These parameters are unfortunately not available for our samples, which are from open sites. To evaluate the presence of DNA, Hoechst 33342 stained fossil eggshell samples were visualized under CLSM. DNA hotspots observed on the periphery of mammillary layer are consistent with shape and size of epithelial fossil cells instead of random shapeless patches, eliminating the possibility of these hotspots being of microbial origin. Modifications in the aDNA isolation procedure of Oskam and Bunce [41] were made at the heating step to prevent complete denaturation of aDNA. Species-specific primers based on *S*. *camelus* were designed to target short fragments. Amplified fragments were further cloned and sequenced. The multiple alignment of sequences confirms the relationship of our samples with other avian species and their identity with *Stuthio camelus*. Molecular phylogenetic tree further revealed the placement of our samples in this clade and a basal position relative to other studied paleognaths, which supports the vicariant speciation theory and other ratites phylogenetic studies.

Morphological studies conducted earlier on these eggshells [6,17,30] indicated their close proximity to *Struthio* species. The present genetic study performed using species-specific primers confirms that the eggshell fragments belong to the *Struthio* species and that these ratites were present in India during the Late Pleistocene. Paleoenvironmental studies indicate favorable environment for survival and expansion of this species in the Indian subcontinent during Late Pleistocene [5]. Currently, ostriches are extinct in India and their sudden extinction coincides with the period of expansion of humans from Africa towards South Asia [5]. There is evidence in the archaeological context for the occurrence of ostrich eggshell beads and crisscross motif patterns on these eggshells with striking similarities to material culture of Africa [36]. These records suggest a connection between the earliest modern humans in southern Asia and their probable ancestors in eastern and southern Africa [36]. However, no conclusive evidence in this regard is available as yet.

The archaeological records of eggshells fragments, beads and rock shelter paintings discovered at Indian sites are consistent with our genetic study, which is the first molecular evidence indicating the presence of ostriches in India. These evidences further endorse the biogeographical movement of ostriches in India when Indo-Madagascar broke apart [3]. Furthermore, this genetic characterization of fossil eggshells is important in studying aspects related to biology, ecology and extinction of this species.

## Supporting Information

S1 Fig Sample collection locations shown on map. (A) Bundi (B) Anjar (C) Chandresal-1 (D) Nagda (E) Runija (F) Khajurna (G) Ravishankar nagar. (Source- Maps at the CIA (public domain): https://www.cia.gov/library/publications/the-world-factbook/index.html).
(TIFF)

S1 Table Estimates of Evolutionary Divergence between Sequences. The number of base substitutions per site from between sequences are shown. Analyses were conducted using the Maximum Composite Likelihood model. The analysis involved 7 nucleotide sequences. Codon positions included were 1st+2nd+3rd+Noncoding. All positions containing gaps and missing data were eliminated. There were a total of 87 positions in the final dataset. Evolutionary analyses were conducted in MEGA6.
(DOCX)
