# Supplementary figures and images for: Ancient DNA Reveals Late Pleistocene Existence of Ostriches in Indian Sub-Continent
Source: PLoS One. 2017 Mar 8;12(3):e0164823. doi: 10.1371/journal.pone.0164823 (PMC5342186; doi:10.1371/journal.pone.0164823)

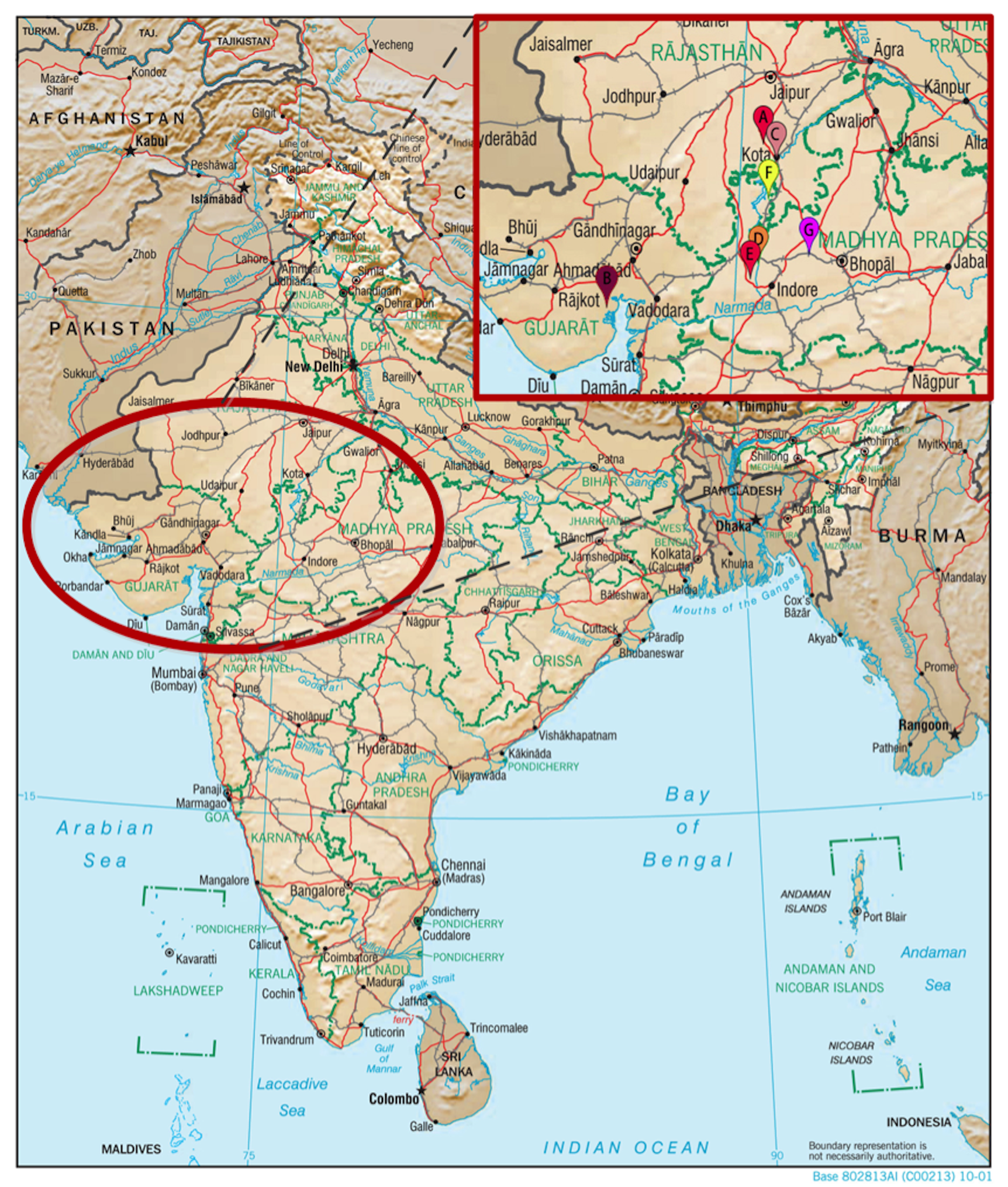

Supplement: S1 Fig — (A) Bundi (B) Anjar (C) Chandresal-1 (D) Nagda (E) Runija (F) Khajurna (G) Ravishankar nagar. (Source- Maps at the CIA (public domain): https://www.cia.gov/library/publications/the-world-factbook/index.html). (TIFF) [file pone.0164823.s001.tiff]
