# Supplementary material for: Ancient DNA Reveals Late Pleistocene Existence of Ostriches in Indian Sub-Continent
Source: PLoS One. 2017 Mar 8;12(3):e0164823. doi: 10.1371/journal.pone.0164823 (PMC5342186; doi:10.1371/journal.pone.0164823)
Supplement: S1 Table — The number of base substitutions per site from between sequences are shown. Analyses were conducted using the Maximum Composite Likelihood model. The analysis involved 7 nucleotide sequences. Codon positions included were 1st+2nd+3rd+Noncoding. All positions containing gaps and missing data were eliminated. There were a total of 87 positions in the final dataset. Evolutionary analyses were conducted in MEGA6. (DOCX) [file pone.0164823.s002.docx]

|  | 1 | 2 | 3 | 4 | 5 | 6 |
| --- | --- | --- | --- | --- | --- | --- |
| 1. Struthio camelus australis (Ostrich) |  |  |  |  |  |  |
| 2. Struthio camelus ultra-conserved element locus chr3 2745 genomic sequence | 0.991 |  |  |  |  |  |
| 3. Apteryx australis mantelli (Kiwi) | 0.974 | 1.404 |  |  |  |  |
| 4. GK/RN/011 (sample) | 0.404 | 1.033 | 1.005 |  |  |  |
| 5. Anomalopteryx | 0.555 | 1.667 | 0.840 | 0.668 |  |  |
| 6. Dromaius novaehollandiae (Emu) | 0.555 | 1.667 | 0.840 | 0.668 | 0.000 |  |
| 7. Rhea Americana (Rhea) | 1.176 | 1.776 | 1.007 | 1.486 | 0.964 | 0.964 |

S1 Table
